# Limited detection of human polyomaviruses in Fanconi anemia related squamous cell carcinoma

**DOI:** 10.1371/journal.pone.0209235

**Published:** 2018-12-27

**Authors:** Tuna Toptan, Marion G. Brusadelli, Brian Turpin, David P. Witte, Jordi Surrallés, Eunike Velleuer, Martin Schramm, Ralf Dietrich, Ruud H. Brakenhoff, Patrick S. Moore, Yuan Chang, Susanne I. Wells

**Affiliations:** 1 University of Pittsburgh, Hillman Cancer Center, Pittsburgh, Pennsylvania, United States of America; 2 Division of Oncology, Cancer and Blood Diseases Institute, Cincinnati Children’s Hospital Medical Center, Cincinnati, Ohio, United States of America; 3 Division of Pathology, Cancer and Blood Diseases Institute, Cincinnati Children’s Hospital Medical Center, Cincinnati, Ohio, United States of America; 4 Department of Genetics and Microbiology, Genetics Department and Biomedical Research Institute of Hospital de les Santes Creus i Sant Pau, Universitat Autònoma de Barcelona, and Centro de Investigación Biomédica en Red de Enfermedades Raras (CIBERER), Barcelona, Spain; 5 Department of Pediatrics, Hospital Neuwerk Maria von den Aposteln, Mönchengladbach, Germany; 6 Department of Cytopathology, Institute of Pathology, Heinrich Heine University, Düsseldorf, Germany; 7 Deutsche Fanconi-Anämie-Hilfe e.V., Unna-Siddinghausen, Germany; 8 Amsterdam UMC, Vrije Universiteit Amsterdam, Otolaryngology - Head and Neck Surgery, Cancer Center Amsterdam, Amsterdam, The Netherlands; Ohio State University Wexner Medical Center, UNITED STATES

## Abstract

Fanconi anemia is a rare genome instability disorder with extreme susceptibility to squamous cell carcinoma of the head and neck and anogenital tract. In patients with this inherited disorder, the risk of head and neck cancer is 800-fold higher than in the general population, a finding which might suggest a viral etiology. Here, we analyzed the possible contribution of human polyomaviruses to FA-associated head and neck squamous cell carcinoma (HNSCC) by a pan-polyomavirus immunohistochemistry test which detects the T antigens of all known human polyomaviruses. We observed weak reactivity in 17% of the HNSCC samples suggesting that based on classical criteria, human polyomaviruses are not causally related to squamous cell carcinomas analyzed in this study.

## Introduction

Fanconi anemia (FA) is a rare DNA repair deficiency syndrome where inactivating germline mutations in FA genes confer susceptibility to bone marrow failure, leukemia and solid tumors, particularly squamous cell carcinoma (SCC) [1–6]. 22 genes encoding FA complementation group (FANC) proteins that contribute to DNA repair have been identified. Analysis of 754 individuals with FA in the International Fanconi Anemia Registry revealed that the cumulative incidence for developing squamous cell carcinoma of the head and neck (HNSCC) is 14% by 40 years of age, compared to 0.038% in the general population [7]. In early studies, the possibility that human papillomavirus (HPV) infection may be associated with SCC was proposed because FA individuals have impaired immune responses that might increase susceptibility to viral tumorigenesis [8–10].

High-risk human papillomavirus (HPV), particularly HPV16, is an established cause of sporadic anogenital and oropharyngeal SCC in healthy and immunosuppressed individuals [11]. HPV replication is limited by the FA pathway and FANCA or FANCD2 deficiency can lead to HPV-associated hyperplastic growth in 3D engineered epidermis [12]. Although several groups have detected the presence of HPV in SCC [13–15], as well as seropositivity and increased prevalence of oral HPV in FA patients [16], a causal association for the high-risk HPV types 16/18 in FA HNSCC remains controversial [17–20].

Since the epidemiology of FA HNSCC suggests a possible viral etiology, other previously unexamined epidermotropic viruses may be viable candidates to cause this tumor type. Human polyomaviruses (HPyV) are a family of DNA tumor viruses distantly related to papillomaviruses. These ~5 kb double-stranded DNA genome viruses encode for tumor (T) antigens that are potential oncoproteins. Only one HPyV, Merkel cell polyomavirus (MCV), is currently established to cause human cancer. MCV is responsible for ~80% of all Merkel cell carcinoma (MCC), a highly aggressive neuroendocrine skin tumor [21]. In all virus-associated tumors, the MCV genome is clonally integrated generally at >1 copies in each tumor cell [22, 23] and express oncogenic viral T antigen proteins. The remaining ~20% of MCC are thought to arise from UV-induced genomic damage and tend to have high mutation burdens [24]. While MCV DNA has been reported to be variably detectable in these latter group of tumors, the calculated viral copy numbers are exceedingly low and are most consistent with coincidental infection by an epidermotropic virus. Several studies have detected low copy numbers (0.00024–0.026 copy/cell) of MCV DNA in oral cavity tumors of immunocompetent patients [25], and in saliva and oral samples collected from healthy individuals [26]. Occasional MCC cases originating from the oral cavity have been reported [27].

Here we investigated whether any of the known HPyVs are commonly detected in FA associated cancers at a level consistent with an etiologic association. We used a pan-polyomavirus immunohistochemistry test (P-PIT) which is a three antibody cocktail that detects T antigens of BK virus, JC virus, WI virus, KI virus, MCV, HPyV6, HPyV7, Trichodysplasia spinulosa virus, HPyV9, HPyV10, HPyV11, HPyV12 and NJPyV [28]. P-PIT is a robust assay which not only determines whether viral T antigen is expressed, but also provides critical localization information to aid in evaluating whether viral detection is associated with tumor causation or merely represents a passenger infection. Recently, we used P-PIT to identify a new rat polyomavirus (RatPyV2) in an immunocompromised rat colony [29] reinforcing the potential of this assay for novel polyomavirus detection and discovery.

## Materials and methods

### Human tissue samples

The Cincinnati Children’s Hospital Medical Center Institutional Review Board (IRB) reviewed the collection of de-identified human tissue samples described here (Study #2011–2934, Epithelial Tumors in Fanconi Anemia). The IRB determined that this study does not meet the regulatory criteria for research involving human subjects, and that ongoing IRB oversight is not required. De-identified fixed and paraffin-embedded FASCC tissues samples were provided by the National Disease Research Interchange (NDRI), USA (FASCC 1–2), the Deutsche Fanconi-Anämie-Hilfe d.V., Germany (FASCC 3–4), the Department of Pathology at Heinrich Heine University, Germany (FASCC 5–16), the Department of Genetics and Microbiology at the University Autonoma de Barcelona, Spain (FASCC 17–20), the Department of Otolaryngology at the VU University Medical Center, Netherlands (FASCC 21–28), and Cincinnati Children’s Hospital (FASCC 29).

### Immunohistochemistry

Cell-pellet microarrays were constructed from HEK293 cells separately expressing the T antigens of each of the known HPyVs as previously described [28]. Cell pellet microarray, tissue sections from FASCC patient and MCV positive and negative MCC control sections were stained according to a previously published protocol using the P-PIT cocktail comprised of primary antibodies PAb416 (1:100), 2t2 (4 μg/ml concentrated mAb), xt7 (4 μg/ml concentrated mAb) or CM2B4 (1 μg/ml mAb) for 1 h at room temperature [28].

### DNA extraction and quantitative PCR analysis

DNA was extracted from tissue samples using QIAmp DNA FFPE Tissue kit according to manufacturer’s recommendations (Qiagen). 100 ng genomic DNA of each sample was used for quantitative PCR analysis performed in triplicates as previously described [22]. Primers amplifying MCV LT antigen (1052–1131 nt; forward: 5’-ctctgggtatgggtccttctca-3’, reverse: 5’-catggtgttcgggaggtatatcg-3’) and internal probe (5’-ccaggcttcagactcc-3’) labeled with FAM and MGB-NFQ quencher (Applied Biosystems) were used. Copy numbers were calculated by generating standard curves of Ct values obtained from serial dilutions of known concentrations of MCV DNA amplified by PCR. RNaseP (Applied Biosystems) was used to determine cell number. qPCR reactions were performed on the Bio-rad CFX96 Real-Time PCR system (Bio-rad) with UNG (+) TaqMan Universal PCR Master Mix II (Applied Biosystems). Amplification reactions of all target genes were performed with the following condition: 50°C for 2 min, denaturing at 95°C for 10 min, 40 cycles of 95°C for 15 sec and 60°C for 1 min. Results were expressed as numbers of viral copies per cell calculated from Ct values of viral and cellular gene standards.

## Results

**Fig 1:**
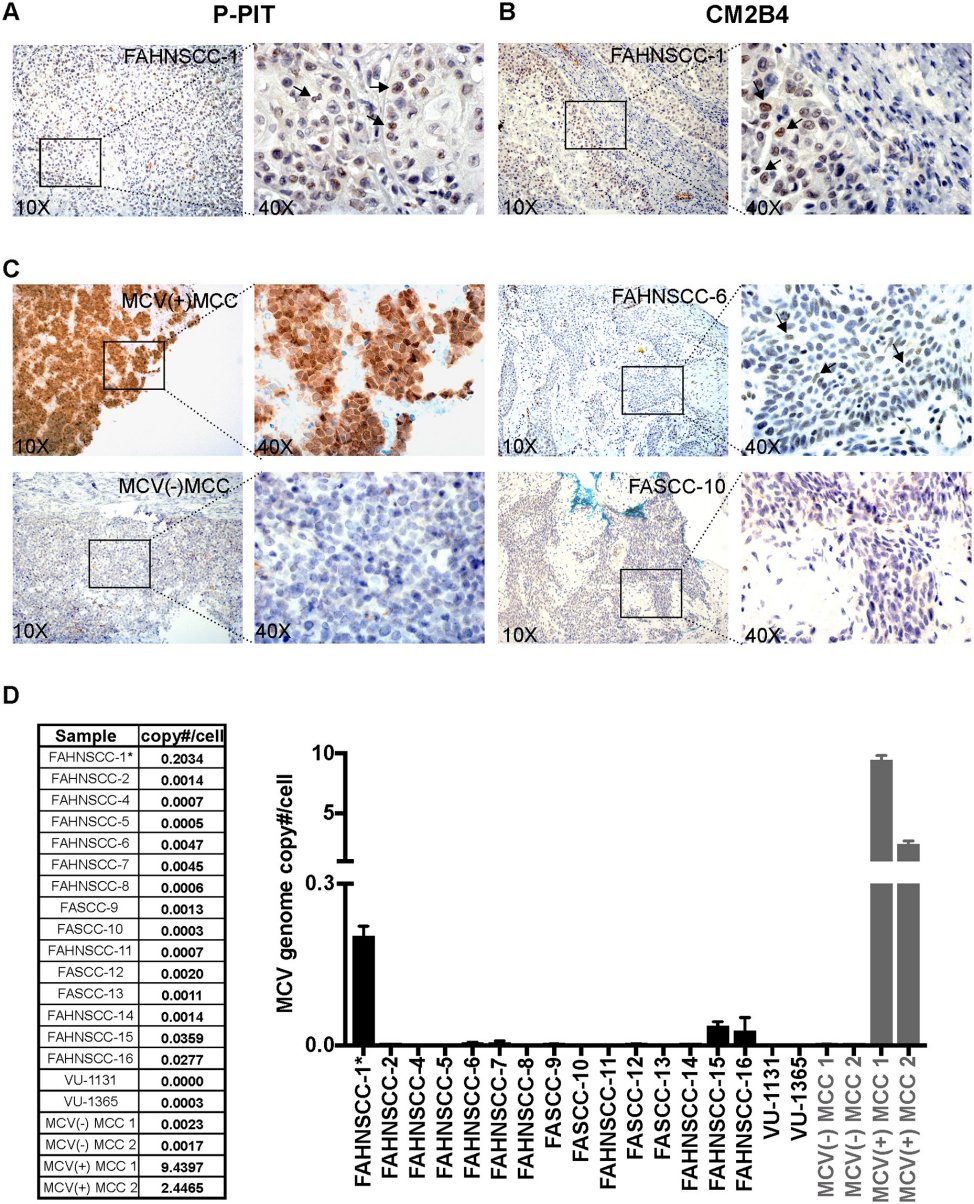
T antigen expression in FAHNSCC. Formalin-fixed paraffin embedded tissue sections from FAHNSCC samples were immunostained with (A) P-PIT antibody-cocktail comprised of PAb416, xt7, or 2t2 or with (B) CM2B4. Black arrows indicate weak nuclear signal detected in the tumor area of these tissues. (C) Left, IHC-staining with CM2B4 of MCV positive (MCV(+) MCC) and negative (MCV(-) MCC) control tissues. Right, IHC-staining with CM2B4 of FAHNSCC-6 and FASCC-10 cases as representative examples for inconclusive (+/-) and negative (-) staining pattern respectively. (D) Detection of MCV genome with quantitative PCR in various FASCC tumor tissues and cell lines. Standard RNaseP copy number was divided by two to determine the cellular equivalent of DNA. FAHNSCC-1* DNA was extracted from the cell line corresponding to the tissue analyzed in Fig 1A and Table 1.

We analyzed twenty-nine FA squamous cell carcinomas by P-PIT. MCV positive and negative MCC tissues along with a cell-pellet microarray comprised of HEK293 cells expressing T antigens of all thirteen known HPyVs were used as controls. A score for each case was assigned as high positive (+++), positive (++), low positive (+), inconclusive (+/-) or negative (-) (Table 1). Out of 29 FASCC samples, three (FAHNSCC-1, 3, and 17) showed weak nuclear staining (+) with the P-PIT antibody-cocktail in tumor cells (Table 1, Fig 1A, left). Staining of these tissues with each antibody alone (pAB416, 2t2, xt7) showed weak reactivity with 2t2, an MCV specific antibody. We verified these IHC results using another MCV LT-specific antibody, CM2B4 (Fig 1B and 1C). Whereas MCV T antigen-positive MCC control tissue showed robust nuclear reactivity in the tumor cells (Fig 1C), reactivity in FAHNSCC tissues was much weaker and inconsistently scattered within the tumor. Some tissues showed cytoplasmic background (cyto bg) staining most likely due to different fixation methods and/or non-specific binding to keratin (Table 1). In FAHNSCC-5, FAHNSCC-8, and FAHNSCC-25 we observed a few cells with strong nuclear staining (1–5 cells/ section) suggestive of passenger infection.

**Table 1:** Summary of T antigen expression status, localization, and tumor collection year of FA-associated tumor samples used in this study.

Tissue samples	Tumor site	Year	IHC staining	
			PPIT	CM2B4/2t2
FAHNSCC-1	Tongue	2010	+	+
FAHNSCC-2	Oral cavity	2015	(+/-)	-
FAHNSCC-3	Larynx	2015	+	++
FAHNSCC-4	Tongue	2016	(+/-)	-
FAHNSCC-5	Metastatic lymph node (Hypopharynx)	2015	cyto bg/ few nuc	ND
FAHNSCC-6	Tongue	2007	-	(+/-)
FAHNSCC-7	Oral cavity	2014	(+/-)	(+/-)
FAHNSCC-8	Oral cavity	2015	cyto bg/ few nuc	ND
FASCC-9	Clitoris	2008	-	ND
FASCC-10	Soft tissue metastasis	2015	-	-
FAHNSCC-11	Hypopharynx	2015	cyto bg/ few nuc	ND
FASCC-12	Anus	2011	cyto bg	ND
FASCC-13	Vulva	2011	cyto bg	ND
FAHNSCC-14	Hypopharynx	2016	(+/-)	+
FAHNSCC-15	Oral cavitiy	2015	(+/-)	-
FAHNSCC-16	Oral cavitiy	2009	-	ND
FAHNSCC-17	Buccal mucosa	1997	+	ND
FAHNSCC-18	Oral Cavity	1997	-	ND
FAHNSCC-19	Hypopharynx	2000	-	ND
FASCC-20	Vulva	2003	-	ND
FAHNSCC-21	Tongue	2011	-	ND
FASCC-22	NA	NA	cyto bg	ND
FAHNSCC-23	Esophagus	2004	cyto bg	-
FAHNSCC-24	Buccal mucosa	1998	cyto bg	-
FAHNSCC-25	Esophagus	2003	cyto bg/ few nuc	(+/-)
FAHNSCC-26	Oral cavity	NA	cyto bg	-
FAHNSCC-27	Metastatic lymph node (Epiglottis)	2014	-	-
FAHNSCC-28	Oral cavity	2011	cyto bg	(+/-)
FAHNSCC-29	Oral cavity (site 1)	2016	cyto bg	ND
	Oral cavity (site 2)	2016	-	ND
MCV(-) MCC 1	Skin	2014	-	-
MCV(-) MCC 2	Skin	2014	-	-
MCV(+) MCC 1	Skin	2016	+++	+++
MCV(+) MCC 2	Skin	2015	++	+++

NA: not available, ND: not determined, cyto bg: cytoplasmic background, nuc: nuclear staining, (+/-): inconclusive, -: negative, +: positive IHC staining.

Due to limited tissue availability, we were only able to obtain DNA from twelve FFPE samples (FASCC5-16), two frozen tumor tissues (FAHNSCC-2 and FAHNSCC-4) and one early passage cell line derived from the FAHNSCC-1 sample (FAHNSCC-1*) (Fig 1D). The FAHNSCC-1* cell line and four FA samples (FAHNSCC6, FAHNSCC7, FAHNSCC15, FAHNSCC16), had MCV copy numbers of >0.0023 copies/cell, which is comparable to copy number determinations from MCV negative MCC control cases. Established FA cell lines (VU-1131 and VU-1365) [19], and all other FA tumor sample DNA had MCV copy numbers below 0.0023 copies/cell. In the majority of FA tumor samples (FASCC-2 through -16) MCV-specific qPCR results were within the expected range for the oral cavity as previously described (an average of 0.026 copies/ cell) [26]. Three log higher MCV DNA copy numbers (2–9 MCV genome copies/cell) were detected in positive control DNA samples extracted from MCV-positive MCC FFPE tissues. DNA from the FAHNSCC-1* line showed a relatively higher copy number of MCV (0.203 copies/cell).

P-PIT and CM2B4 staining were weakly reactive in FAHNSCC-3, however we were not able to obtain sufficient amounts of DNA to pursue further analysis. For most of the tissues analyzed, we were unable to perform PCR analysis using overlapping primers to walk the genome due to insufficient DNA quality, a limitation for DNA extracted from formalin fixed, paraffin-embedded tissues. We did not observe any correlation with CM2B4 staining and MCV copy numbers detected in FA tumor cases other than FAHNSCC-1.

## Discussion

FA is characterized by extreme susceptibility to bone marrow failure and SCC of the head and neck, esophagus, anogenical tract and skin. HPV, and more recently polyomavirus infections, have been linked to these tumor types in the general population. The association of HPV with HNSCC in FA remains controversial due to variable results in the detection of HPV in these tumors by different laboratories. The goal of the current study was to determine whether human polyomaviruses are causally associated with FASCC by using immunohistochemical detection and localization of polyomavirus T antigens which is correlated, in cases with available DNA, by PCR analysis.

Seventeen percent of the FAHNSCC showed weak reactivity using pan-PyV antibodies. The weak positivity of some of the specimens may suggest a hit-and-run mechanism for carcinogenesis similar to a scenario proposed for HPV in FA [20]. This scenario was based on key studies by the Lambert laboratory where sustained SCC growth in *Fancd2* knockout mice occurred even in the absence of the viral E7 oncogene, perhaps due to virally induced DNA damage accumulation and rapid selection for oncogene-independent tumor growth [30]. However, technical reasons including variations in preservation/processing of tissues might also explain these observations. Serological studies might reveal a distinct pattern or level of antibody reactivity for certain polyomaviruses in FASCC patients, similar to what is seen with the high anti-VP1 mean titer levels in MCV positive MCC patients compared with MCV negative MCC patients [31]. However, serologic assays have not been developed for all human polyomaviruses underscoring the utility of P-PIT. Moreover polyomavirus infection tends to be ubiquitous [32]. For example, MCV infection occurs early in childhood and is widespread [32] with a seroprevelance of 35–50% in children <10–15 years-old [32, 33]. Therefore serological profiling alone may not be sufficient to establish a direct link between a particular human polyomavirus and tumorigenesis in FA patients.

To our knowledge, this is the first study investigating a potential causal association between HPyVs and FAHNSCC patients. By the classical model of viral tumorigenesis, viruses associated with human cancers effect a tumor phenotype through the expression of one or a combination of viral products. The corollary to this is that the viral genome or a part of the viral genome that elaborates these viral onco-molecules must be in each and every tumor cell. Because FA is a rare syndrome, our data is limited to a small number of patient samples that were collected across multiple institutions within the past 21 years. If MCV is etiologically linked to a small subset of FAHNSCCs, then this study may be underpowered to detect such an association. Nevertheless, MCV genome copies detected with qPCR and the immunostaining pattern with P-PIT and MCV specific CM2B4 suggest that based on conventional criteria, neither MCV nor other known human polyomaviruses are causal pathogens for FA-related SCCs.
